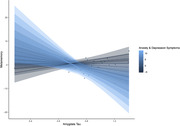# Influence of tau pathology and anxiety and depressive symptoms on metamemory in cognitively normal older adults

**DOI:** 10.1002/alz.087366

**Published:** 2025-01-09

**Authors:** Jennifer L. Crawford, Alex Adornato, Johanna Matulonis, Jacob M. Hooker, Anne S. Berry

**Affiliations:** ^1^ Brandeis University, Waltham, MA USA; ^2^ Martinos Center for Biomedical Imaging/Harvard Medical School/Massachusetts General Hospital, Charlestown, MA USA

## Abstract

**Background:**

The ability to accurately discern memory function (i.e., metamemory) decreases with age and is further diminished in Alzheimer’s disease (AD). As such, changes in metamemory may be a sensitive behavioral indicator of pre‐symptomatic AD.

**Method:**

Cognitively normal older adult participants (N=48) completed a neuropsychological battery consisting of episodic memory tasks (California Verbal Learning Task, Face‐Name Associative Memory Exam) and self‐reported measures of metamemory (Multifactorial Memory Questionnaire) and anxiety and depressive symptoms (Geriatric Depression Scale, Penn State Worry Questionnaire, Mood and Anxiety Symptom Questionnaire). Z‐scores from each measure were combined into three separate composite scores: memory ability, metamemory, and anxiety and depressive symptoms. All participants also had a tau PET scan ([18F]MK‐6240) completed within 12.7 (SD = 11.6) months of neuropsychological testing in which standardized uptake value ratios (SUVR) referenced to the inferior cerebellar gray matter were calculated using Freesurfer‐defined ROIs for Braak I, Braak II, metatemporal, and amygdala regions of interest (ROIs). Linear regression models were used to test for the contribution of anxiety and depression symptoms, tau pathology, and memory ability on metamemory; education, age, and sex were entered as covariates in all models.

**Result:**

Behavioral analyses suggest that anxiety and depression symptoms (t = ‐2.1, p=0.046), but not memory ability (t = 0.4, p=0.698), were related to metamemory. Interestingly, tau pathology helped to explain additional variance in the relationship between metamemory and anxiety and depressive symptoms. Specifically, amygdala tau interacted with anxiety and depression symptoms to predict metamemory (t = ‐2.61, p=0.013), when controlling for age, sex, education, and memory ability, such that participants with both high levels of anxiety and depressive symptoms and high levels of amygdala tau pathology had lower self‐reported metamemory (Figure 1). This relationship was not observed in Braak I, Braak II, or metatemporal ROIs (ps > 0.07).

**Conclusion:**

Together, these results suggest that depressive and anxiety symptoms are an important metric to consider when examining the relationships between tau pathology and metamemory. Further, the results underscore that mechanisms supporting metamemory are likely distinct from those underlying memory ability and each merit unique consideration towards understanding their relationship to AD pathology.